# Remarks, Gleanings, and Extracts

**Published:** 1874-06

**Authors:** R. C. Word

**Affiliations:** Decatur, Georgia


### REMARKS, GLEANINGS AND EXTRACTS

BY R. C. WORD, M.D., OF DECATUR, GEORGIA.

*Dysentery, Ergot in.*—The Cincinnati *Medical News* states that M. Luton, of Rheims, had found, in giving *ergot* to a female patient, who was suffering at the same time from utering hemorrhage and dysentery, that both affections were promptly relieved. This led to the use of ergot in simple dysentery. It proved an efficacious remedy. Three grammes (about 45 grains) were given in the course of a day, in doses of 7½ grains. Two or three days generally sufficed for a cure. It appeared to attack not only the hemorrhagic element, but the whole disease, and the mucous secretions; the griping colic and fever were equally relieved. We have not tried the ergot in this disease. For most cases of dysentery we prefer the saline treatment, conjoined with anodynes and revulsives. We usually commence the treatment with a large sinapism to the abdomen, to be followed by warm formentations and the following internal remedy: ℞. Sulphate magnesia, two teaspoonsful, water,

℥iij; tinc. opi. deodorati, ʒj. M. Give one teaspoonful every two hours until the change in the discharges show that it has acted; then continue once every four hours. In most cases the disease will be converted into a mild diarrhœa by this plan in twenty-four to forty-eight hours, when it may be arrested by opiates. If the patient is bilious, ten to fifteen grains *chalk and mercury* should be given before commencing the saline mixture; and, if there is malarial complication, quinine should be given every morning.

*Blood-letting in Peurperal Convulsions.*—It is a great point in modern practice to descant on the empirical use of the lancet in former days, whilst there is quite as much empiricism in the unqualified disuse of it by modern extremists. In a discussion in the New York Pathological Society (*Medical Record*, Jan. 2,) a member condemned the practice in puerperal convulsions, saying "he believed in chloroform in all cases; it was the first thing he thought of, and he had never found it to disappoint him." Dr. Small referred to the suffused face, bounding pulse and pain in the head, with incipient paralysis, as denoting a condition not to be permanently relieved by chloroform, but requiring loss of blood. Dr. Briddon had seen a large number of cases of pueperal convulsions, and whenever he had treated them by venesection he had been uniformly successful. His point was to use the remedy early. In some cases when chloroform had been used first, with the effect of arresting the convulsions for a short time, venesection gave permanent relief, speedily restoring consciousness.

[Bleed and give bromide of potash freely. We have tried this successfully.—Ed.]

*A Dangerous Paper.*—The green paper used to wrap about lozenges, sold in shops, railroad cars, and on street corners, has long been suspected to contain arsenic; and, with the view of ascertaining the facts by analysis, we recently purchased a roll of lozenges covered with this paper. A qualitative examination of the paper afforded all the characteristic reactions for arsenic and copper. The wrapper contained twenty square inches of paper. Of this, sixteen were taken for quantitative analysis. The result of the examination showed that this portion contained .1516 grams, or 2.34 grains of metallic arsenic. This is equivalent to 2.94 grains in the whole of the wrapper, a quantity sufficient to destroy life in an adult person. Children in all parts of the country are allowed to purchase the lozenges covered with this poisonous paper, and the rolls are often put into the hands of infants as a plaything. As everything goes into the mouth of young children, it is easy to see that no more dangerous substance can pass into a family than these packages of confectionery. It is quite probable that instances of poisoning have occurred from this cause, which have been of a serious or fatal character. There should be laws prohibiting the use of poisonous papers for any purpose.—*Boston Journal of Chemistry.*

*Podalic Version.*—Dr. Alexander Milne, corresponding member of the Gynæcological Society of Boston, in an article to the London *Lancet*, states that the operation of podalic version, where the parts have become swollen and dry, and the uterus has contracted firmly around the fœtus—a condition of things which has been generally regarded as contraindicating, or at least making very doubtful, the practicability of the operation of turning—he has found the best means to promote relaxation is the use of tart. antimony or lobelia conjoined with chloroform. Give a grain of the antimony and twenty-five drops of the tincture of lobelia; after the lapse of twenty minutes, place the patient under the influence of chloroform, and in a large proportion of cases the uterus will relax, the hand find ready entrance, and version become possible. The same means will succeed in shoulder presentations, even where the arm has been protruding for hours. He recommends the use of the left hand in all cases of podalic version.

*Cinquo-Quinine.*—We have tested this new preparation in a number of cases of intermittent fever, with satisfactory results. In one severe case of remittent fever in the country, which we did not expect to visit again, we left ten powders, four grains each, with instructions to take one every three hours continuously until all were taken. The experiment was successful, the patient having recovered without further medication.

Its cheapness recommends it to the country practitioner who is compelled to give away large quantities of drugs. It sells at about half the cost of the sulphate.

It is said to contain all the alkaloids of Peruvian bark, and to possess not only the properties of the sulphate of quinine, but other principles equally valuable, and giving it a wide therapeutical application.

*Anthemis Cotula.*—The above plant, being the common "*May weed*," or "*Dog Fennel*," is too well known in this country to require description. The flowers of the plant resemble those of the imported chamomile, and are said to possess similar properties. In domestic practice, they have been employed as an emenagogue, and a tea of the leaves has been found efficacious in colic and dysentery. In large doses, it is said to be a prompt emetic. But the property to which we would specially draw attention in this weed is its power of blistering the skin. The green leaves bruised and applied to the skin will produce epaspastic effects equal, if not superior, to cantharides. If kept on long after vesication commences, they will generally make a painful and troublesome surface sore, covered with pustules, which require several days to heal—a property which may be turned to good account in cases demanding a powerful and permanent counter-irritation.

*New Plan of Extracting Bodies from the Ear.*—Dr. Loewenberg, of Paris, describes a new plan for extracting solid bodies from the ear. A very small brush is made by rolling, and fixing a narrow strip of old linen around a thin wooden handle (a match, for instance), and unraveling its free border to the length of a quarter of an inch. The end of the so-obtained fringe is dipped into a warm and very concentrated solution of glue, applied to the visible part of the foreign body—or rather the operator leans it against the body by letting it glide very softly, and without exercising any pressure, over it. Previous to the application, the patient seats himself comfortably in an arm-chair or on a sofa, and inclines his head toward the healthy ear. He remains in this posture for three-quarters of an hour to an hour after the introduction of the agglutinated brush. This time past, consolidation is generally accomplished, and the foreign body can be extracted by gentle pulling at the brush.—*Boston Journal of Chemistry.*

*A Medical Witness Answers Back.*—In the famous Wharton trial which lately took place in Baltimore, the prosecuting attorney, finding that the testimony of Dr. Warren, a witness on behalf of Mrs. Wharton, did not suit his purposes, resorted to the common expedient of badgering the witness, with the following result:

*Attorney.*—A doctor ought to be able to give an opinion of a disease without making mistakes.

*Doctor.*—They make as few mistakes as lawyers.

*Attorney.*—But doctors' mistakes are buried six feet under ground; a lawyer's are not.

*Doctor.*—No; they are sometimes hung up six feet above ground.

*Vomiting of Pregnancy.*—Dr. Atthill, in the *Medical Press and Circular*, says that the hypodermic injection of morphia occasionally controls the vomiting met with in pregnancy, or that which sometimes follows severe cases of *post-partum* hemorrhage. The formula which he now adopts for the solution to be injected subcutaneously is the following:

℞.—Acetatis morphiæ, gr. viij.; liquoris atropiæ, m. xlviij.; glycerini, m. iv.; aquæ ad., ʒ iv. Fifteen drops of this solution contain half a grain of the acetate of morphia, and about the fortieth of a grain of atropia.—*Journal Materia Medica.*

